# Prevalence of *Borrelia afzelii* and *Borrelia garinii* Genospecies in *Ixodes ricinus* Ticks Collected from Three Regions in Bulgaria

**DOI:** 10.3390/pathogens14121240

**Published:** 2025-12-04

**Authors:** Iskren Stanilov, Alexander Blazhev, Borislava Chakarova, Spaska Stanilova

**Affiliations:** 1 Department of Hygiene, Epidemiology, Microbiology, Parasitology and Infectious Diseases, Medical Faculty, Trakia University, 6000 Stara Zagora, Bulgaria; iskren.stanilov@gmail.com (I.S.); borislava.chakarova@trakia-uni.bg (B.C.); 2 Department of Biology, Medical University—Pleven, 5800 Pleven, Bulgaria; yalishanda9@gmail.com; 3 Department of Molecular Biology, Immunology and Medical Genetics, Medical Faculty, Trakia University, 6000 Stara Zagora, Bulgaria

**Keywords:** *Borrelia burgdorferi*, Lyme borreliosis, nPCR, molecular identification, genospecies

## Abstract

The tick species *Ixodes ricinus* is the most widely distributed throughout Europe and serves as the primary vector for the spirochete *Borrelia burgdorferi* sensu lato (s.l.), which is responsible for Lyme borreliosis. The present study evaluated the prevalence of *B. burgdorferi* (s.l.) in *I. ricinus* ticks using nPCR amplification. Ticks were collected from three geographical regions in Bulgaria: the Black Sea Coast in the east, the Pleven region in the north, and the Stara Zagora region in the central south. This study focused on urban and peri-urban areas, including heavily traveled trails in parks. The results indicated statistically significant differences in infection rates, with the highest percentage of infected ticks found in Pleven (52.34%), followed by Stara Zagora (35.46%) and the lowest on the Black Sea Coast (23.08%). Furthermore, we conducted genospecies molecular identification of *B. afzelii* and *B. garinii* by PCR amplification of genospecies-specific nucleotide sequences in the 16S rRNA gene. The lowest prevalence of *B. afzelii* was recorded in Stara Zagora at 10.64%, followed by the Black Sea region at 12.5%, while the statistically highest frequency was observed in Pleven at 21.03%. The prevalence of *B. garinii* among the total number of *I. ricinus* ticks was greatest in Pleven at 15.89%, followed by the Black Sea region at 8.65%, and the lowest in the Stara Zagora region at 4.96%. Statistically significant differences were found only between Pleven and Stara Zagora (*p* = 0.002), but no significant differences in infection rates were observed between adults and nymphs in the regions examined. Overall, the prevalence of *B. afzelii* in each region exceeds that of *B. garinii*, and when considered collectively for Bulgaria, the frequency of *B. afzelii* (15.9%) is higher than that of *B. garinii* (10.89%) in infected ticks. In a controlled epidemiological context, managing the population of *I. ricinus* infected with *B. burgdorferi* s.l. can mitigate the health burden of Lyme disease, although this preventive strategy may not guarantee complete protection.

## 1. Introduction

The tick genus *Ixodes* is the most widely distributed in Europe and Bulgaria, and it holds significant medical importance as a vector for various tick-borne zoonotic diseases, primarily the spirochete *Borrelia burgdorferi* sensu lato (s.l.), which causes Lyme borreliosis (LB). The likelihood of developing LB in humans depends on the rate of infection of ticks with *Borrelia burgdorferi* s.l. [1,2]. *Ixodes ricinus* (*I. ricinus*) ticks can acquire infection during a blood meal on an infected reservoir host, starting in the larval stage, and can subsequently transmit the spirochete pathogen to nymphs and adults. The mechanisms underlying the maintenance and transmission of *B. burgdorferi* s.l. in natural cycles involve complex relationships between the vector, multiple vertebrate reservoir hosts, and the genotype of the spirochete [3]. Humans are typically accidental hosts, and for the majority of cases, they serve as dead-end hosts.

The species complex, known as *Borrelia burgdorferi* sensu lato, includes the spirochetes that cause LB in humans and some animals. The enzootic cycling of *B. burgdorferi* s.l. between *I. ricinus* and its competent vertebrate hosts is greatly influenced by the local environment. The complex currently consists of 22 recognized genospecies, distributed worldwide with varying frequencies [4,5].

Ticks of the genus *Ixodes* inhabit mainly warm and temperate regions, but some species can also withstand cold weather. Their successful survival depends on optimal temperature and humidity in their habitats, as well as on the availability of suitable competent hosts, including in urban areas [6,7,8]. The expansion of *I. ricinus* populations and escalated incidence of tick bites are tending to intensify across Europe mainly due to anthropogenic factors and climate changes with shorter winter periods [9]. Over the past 20 years, the expansion of their geographic areas, along with the growing number of tick-borne diseases, has demonstrated their significant impact on human health [10,11,12]. Moreover, ticks have now widely spread in urban green areas, including city parks, gardens, and inter-urban and peri-urban forest areas, resulting in an increased number of tick bite incidents for humans and pets, which can lead to a higher tick-borne disease rate [13,14,15]. The identification of tick-borne pathogens and preventive measures are crucial for controlling tick-borne diseases and preventing their spread. Epidemiological surveys can enhance prevention measures, such as using chemical acaricides and other methods to control tick populations. By focusing on the population of *I. ricinus* infected with *B. burgdorferi* s.l., it is possible to mitigate the impact of LB, even if full protection cannot be assured.

An epidemiological study of Lyme Borreliosis in Bulgaria from 2009 to 2018 indicates that northern regions present a significantly higher risk, showing approximately 9.4 times higher incidence rates compared to southern regions, as determined through linear regression analysis [16]. Also, a recent nationwide study on the LB seroprevalence across different regions of Bulgaria found rates that ranged from 0.0% to 20.0%, with the highest rates in Northern provinces reaching up to 20% [17]. Based on these data, we chose one North region, Pleven city park Kaylaka, one South region, Stara Zagora city park Metodiy Kusev, and one more region, close to the Black Sea.

Gladnishka et al. examined 10,907 ticks removed from patients at the National Center for Infectious and Parasitic Diseases between 2016 and 2021. The findings revealed that 92–96% of the ticks were of the species *I. ricinus* [18]. Although there are no officially published data on changes in the tick populations in Bulgaria over the last decade, according to data from the National Center for Infectious and Parasitic Diseases, an increasing number of tick bites has been reported. This trend indirectly suggests an increase in both the distribution and population of ticks.

Research on the infection rate of *I. ricinus* ticks with *B. burgdorferi* s.l. in Bulgaria is limited, primarily focusing on gardens and wooded green spaces in Sofia. Additionally, there is a lack of comprehensive distribution analysis regarding the genospecies of *B. burgdorferi* in Bulgaria. To date, only one study has been published, which examined 246 ticks collected by flagging vegetation in the wooded area of the Sofia region in May 2005 [19].

In the present study, three geographic regions of Bulgaria were included: the Black Sea Coast (eastern part), the Pleven region (north), and the Stara Zagora region (central south). The ticks examined in this study were collected from urban and peri-urban areas, such as public parks and green spaces, which are frequently visited by humans and pets. While these areas provide valuable insights into the epidemiology of LB in environments with high human exposure, they may not fully represent the epidemiological dynamics of *Borrelia burgdorferi* s.l. in rural or less populated regions of Bulgaria.

We examined the prevalence of *B. burgdorferi* s.l. in collected *I. ricinus* ticks by nPCR amplification of two target genes. In addition, we performed genospecies-specific molecular identification of *B. afzelii* and *B. garinii* by PCR amplification of genospecies-specific nucleotide sequences in the *16S rRNA* gene.

## 2. Materials and Methods

### 2.1. Sample Collection

**Figure 1:**
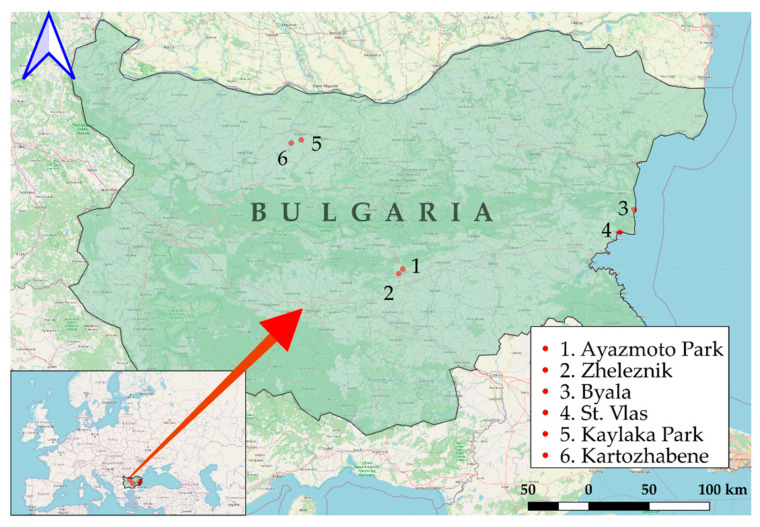
The locations of regions for tick collection. The Stara Zagora region in Central Bulgaria, including Ayazmoto Park (1) and Zheleznik (2). The Black Sea Coast region of Eastern Bulgaria, including the towns of (3) Byala and Sveti Vlas (4). The Pleven region in Northern Bulgaria, including Kaylaka park (5) and village Kartozhabene (6) [20].

The ticks were collected from three geographic regions in Bulgaria between 2020 and 2022: Stara Zagora (Metodi Kusev Park and Zheleznik), Black Sea Coast (Byala and Sveti Vlas), and Pleven (Kaylaka Park, and Kartozhabene) (Figure 1). Geographic mapping was conducted using Quantum Geographic Information Systems (QGIS version 3.18.0, QGIS Development Team, GNU General Public License, Essen, Germany) with the World Geodetic System 1984 (WGS 84) as a standard of coordinate referencing.

The first studied area was in central-southern Bulgaria, specifically around the city of Stara Zagora. The primary site for tick collection was Metodi Kusev (Ayazmoto) Park, where samples were gathered from seven locations, including alleys and meadows suitable for picnics. The second site was the grassy inter-block areas in the district “Zheleznik” (42.4145° N, 25.5886° E), a suburb of Stara Zagora adjacent to a forested region, with an average altitude of 250.0 ± 17.3 m.

Metropolitan Metodiy Kusev Park, better known as Ayazmoto, is one of the highest points around the city of Stara Zagora, located at 42.4399° N, 25.6205° E. The park covers an area of about 320 hectares (3200 decares) north of the city and is rich in wildlife, with an altitude that varies from 260 to 430 m at its highest parts. It is part of the mountainous share of the Sredna (Sarnena) Gora and is forested with over 150 unique tree species for Bulgaria, imported from Mediterranean countries, the most specific of which are the Aleppo pine (*Pinus halepensis*), oak (*Quercus infectoria*), persimmon (*Diospyros kaki*), laurel tree (*Laurus nobilis*), and ginkgo (*Ginkgo biloba*). The main animal species are Northern white-breasted hedgehog (*Erinaceus roumanicus*), European pond turtles (*Emys orbicularis*), Balkan terrapins (*Mauremys rivulata*), squirrels (*Sciurus vulgaris*), roe deer (*Capreolus capreolus*), European badger (*Meles meles*), and a large number of synanthropic rodents, birds, and reptiles, which are potential hosts for both ticks and the pathogenic microorganisms spread by them. The park has numerous grasslands, alleys, and paths, some of which are like green tunnels; hence, it is a preferred location for walking and recreational activities among residents and visitors alike. Due to its popularity, it was selected as a site for the collection of ticks for this scientific research. The findings from this park were compared with the infection rate obtained from the corresponding Kaylaka Park around Pleven.

In 2022, a total of 185 ticks were collected from vegetation in both urban and rural areas of the Stara Zagora region. The main collection points included Cypress Alley, the path leading to the summer theater, and the nearby green playground, as these locations are among the most frequently visited areas in the urban environment. Tick sampling was conducted between March and June under optimal meteorological conditions, specifically during daylight hours, when ambient temperatures ranged between 15 and 25 °C, with no precipitation and wind speeds below 4 on the Beaufort scale. To collect questing ticks, a white flannel cloth with a 1 m^2^ contact area attached to a handle was dragged along low-lying vegetation.

The second region studied was located along the Black Sea Coast of Eastern Bulgaria, specifically around the towns of Byala (42.8807° N, 27.8969° E, average altitude: 42.0 ± 12.1 m) and Sveti Vlas (42.7099° N, 27.7727° E, average altitude: 20.0 ± 17.3 m). Byala and St. Vlas are seaside resorts, located on the Black Sea Coast, that are visited by many people, especially during the summer season. This area encompasses the mountainous section of the coast, where the Balkan Mountain (Stara Planina) meets the Black Sea, and has been described in detail in our previous research [20]. During the period of 2021–2022, a total of 255 ticks were collected from stray dogs in Byala and from domestic dogs at private veterinary clinics in Sveti Vlas. The engorgement status of ticks removed from dogs ranged from unengorged to fully engorged.

The third studied region is located in Northern Bulgaria around Pleven and includes Kaylaka Park (43.3869° N, 24.6231° E, average altitude: 147 ± 38.4 m) and the village of Kartozhabene (43.3564° N, 24.5332° E, average altitude: 153.0 ± 14.4 m). Kaylaka Park is situated on the outskirts of Pleven city and serves as a popular destination for walks and leisure activities, attracting numerous visitors each day. A total of 214 ticks were collected in 2020 and 2021 from vegetation by flagging in urbanized and wild areas in the Pleven region, as previously described in detail [21].

The collected ticks were placed separately in 1.5 mL Eppendorf tubes with a safety lock (Eppendorf AG, Hamburg, Germany) and transported to the laboratory of the Department of “Molecular Biology, Immunology and Medical Genetics” at the Medical Faculty, Trakia University, for species identification and testing for pathogens.

In the laboratory, all ticks underwent taxonomic identification, developmental staging, and sex determination. We identified tick species by examining various morphological features, including the length of the palps relative to the basis capituli, the shape of the basis capituli, the presence of festoons, specific patterns on the dorsal shield, the presence of eyes, the shape of the anal groove, and the shape of the coxae. This identification was conducted using a stereomicroscope (Olympus SZ4045, Olympus American Inc., Melville, NY, USA), following the morphological criteria described by Georgieva and Gecheva, 2013 [22] and Estrada-Peña et al., 2018 [23]. The data obtained from different areas are presented in Table 1.

### 2.2. DNA Extraction and Polymerase Chain Reaction (PCR) for Molecular Identification of Borrelia burgdorferi s.l.: Borrelia afzelii and Borrelia garinii

Genomic DNA was isolated from all collected *Ixodes ricinus* ticks (*n* = 459), using the animal tissue genomic DNA purification mini prep kit (Gennaxxon bioscience, Ulm, Germany), following the manufacturer’s instructions. Each tick was homogenized with 100 µL of buffer and placed in a 1.5 mL tube, to which 275 µL of the same buffer was added. An amount of 75 µL of elution buffer was used in the final step of purification. A spectrophotometer with a 0.5 mm microvolume port, model Biodrop Ulite+ (Biochrom, Cambridge, UK), was used to measure DNA concentration and purity (A260/A280nm was between 1.7 and 2.0). The DNA extracted was stored at −70 °C until the PCR analysis was performed.

To detect the presence of the *Borrelia*’s DNA, we performed nested PCRs (nPCR) targeting two specific genomic nucleotide sequences: non-coding *5S-23S rRNA* intergenic sequences and a protein-coding gene for Flagellin B (*FlaB*). The sequences of primers used and the parameters used for PCR amplification are described in detail in our previous publication [19]. As a positive control, we used purified genomic DNA (isolated from *Borrelia burgdorferi*; strain B31 [ATCC^®^35210D5™], supplied by LGC, Hannover, Germany), diluted to a concentration of 1 ng/mL for PCR amplification. The reagents used were supplied by Thermo Fisher Scientific (Waltham, MA, USA) and Metabion GmbH (Planegg, Germany). A GeneAmp PCR System 9700 from Applied Biosystems (Foster City, CA, USA) was used to perform the polymerase chain reaction.

All DNA samples, positive for at least one gene according to nPCR, were subsequently tested with a second PCR targeting genospecies-specific sequences in the *16S rRNA* gene. For each PCR run, the 20 µL reaction mixture contained 2 µL of 10XPCR buffer, 1.5 mM MgCl_2_, 0.2 mM each of dNTPs, 0.25 µM each of primers, 1U Taq DNA polymerase, and 3 µL of DNA. The primer sets used for *Borrelia afzelii* (*B. afzelii*) and *Borrelia garinii (B. garinii)* identification are presented in Table 2 and have been described previously by Santino et al., 2008 [24].

The annealing temperature was established by gradient PCR performed in an AERIS PCR system (Esco, Singapore). The amplification program was as follows: 95 °C for 5 min; 35 cycles of 95 °C for 30 s, annealing T for 30 s, and 40 s at 72 °C, and a final extension at 72 °C for 5 min.

All PCR products were analyzed using 1.5% agarose gel electrophoresis stained with ethidium bromide, and DNA Ladder (by 100 bp) was applied for evaluation of the obtained product size. The results of the PCR amplification were viewed under UV light and were archived using EasyWin32 software (Herolab; Wiesloch, Germany).

### 2.3. Statistical Analysis

The frequency of *Borrelia burgdorferi* s.l. is reported as counts and as a percentage of the total number of *I. ricinus* ticks, categorized by developmental stage and sex, across the three studied regions. The prevalence of *B. afzelii* and *B. garinii* is expressed as a percentage of *Borrelia burgdorferi* s.l.-positive *I. ricinus* ticks, as well as a percentage of the total number of *I. ricinus* ticks in the regions examined. It should be noted that the prevalence of the individual genospecies, *B. afzelii* and *B. garinii*, was calculated as a percentage of the *B. burgdorferi* s.l.-positive ticks, meaning that co-infected (double-positive) ticks were counted in the prevalence figure for each respective genospecies. This methodology allows for a complete assessment of the circulation of each pathogen, as the sum of the individual genospecies may therefore exceed the total number of *B. burgdorferi* s.l.-positive ticks. To analyze differences in tick infection prevalence between sexes and localities, a nonparametric Chi-square test was conducted, accompanied by a 95% confidence interval (95% CI). A reliability analysis using the kappa statistic was performed to evaluate consistency between nPCR tests for two genes. The results are presented as a kappa coefficient (k) with a 95% confidence interval (95% CI). Differences were considered significant if the *p*-values were less than 0.05.

## 3. Results

### 3.1. Prevalence of Borrelia burgdorferi s.l. in I. ricinus Ticks Collected from the Studied Regions

The data obtained from nPCR for the molecular identification of *B. burgdorferi* s.l. are presented in Table 3. In our study, we focused on two genomic targets: the intergenic spacer located between the 5S and 23S rRNA genes (*5S-23S rRNA*) and the gene encoding the flagellin B protein (*FlaB*).

We found no significant differences in the percentage of positive DNA samples for these two genes. The results for the positive samples tested with nPCR for both genes had substantial agreement, supported by a significant kappa coefficient (κ) of 0.785 (*p* = 0.001, 95% CI (0.703–0.868)), with a level of agreement above 92.25%. That is why samples were classified as positive if they demonstrated DNA amplification for at least one of the tested genes.

The highest prevalence of infected ticks of the species *I. ricinus* was reported for the Pleven region, followed by Stara Zagora, and the lowest rate of infected ticks was observed in the Black Sea region. The difference in prevalence between Pleven (52.34%) and Stara Zagora (35.46%) is statistically significant (*p* = 0.002; χ^2^ = 9.757; OR = 0.500; 95% CI: 3.23–77.5%). In the same direction are the differences between infected ticks for Pleven and the Black Sea region, where the total prevalence established from the tested ticks is 23.08% (*p* < 0.001; χ^2^ = 24.479; OR = 0.273; 95% CI: 16.1–46.4%). The differences in the prevalence of infected ticks between Stara Zagora and the Black Sea region are also significant (*p* = 0.037; χ^2^ = 4.354; OR = 1.832; 95% CI: 10.3–32.4%). For all the studied areas, higher prevalence was reported for female compared to male ticks, but the differences were not statistically significant. The prevalence of infected adult *I. ricinus* ticks is significantly higher in the Pleven region (53.65%) than in the Stara Zagora (34.29%) and Black Sea (22.99%) regions. In addition, no significant differences in prevalence between adults and nymphs were found in the examined regions.

When comparing the two city parks, the prevalence of infected ticks is higher in Kaylaka Park in Pleven (52.97%) than for Metodiy Kusev Park in Stara Zagora (41.98%). However, the differences are not statistically significant (*p* = 0.114; χ^2^ = 2.79; OR = 0.642; 95% CI: 38.2–108.1%).

### 3.2. Prevalence of Borrelia afzelii and Borrelia garinii in I. ricinus Ticks Collected from the Studied Regions

**Figure 2:**
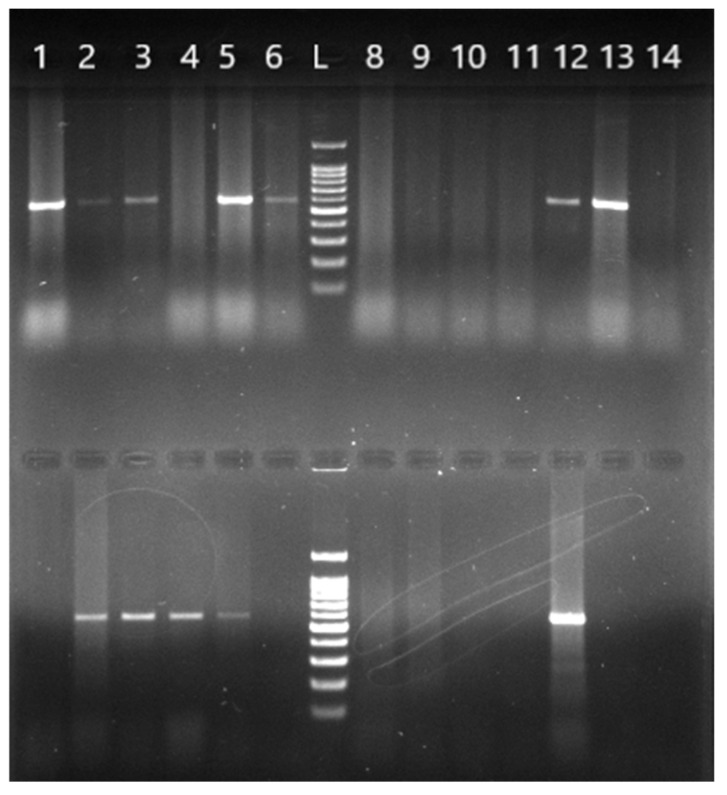
Agarose gel electrophoresis of the PCR products from amplification of the *16S rRNA* gene using genospecific primers. A 591 bp amplicon specific for the species *B. afzelii* (**top row**) and 574 bp amplicon specific for *B. garinii* (**bottom row**), analyzed on a 1.5% agarose gel. The same DNA isolates (1–14) were used in both rows. L—molecular nucleotide marker from 100 to 1500 bp; the first band is 100 bp and the solid band indicates 500 bp.

*B. burgdorferi* s.l.-positive ticks were further analyzed by molecular identification using PCR amplification with genospecies-specific primers, with the results shown in Figure 2.

The distribution of *B. afzelii* and *B. garinii* genospecies in *I. ricinus* ticks from the studied regions is presented in Table 4. Overall, for each region, *B. afzelii* was more prevalent than *B. garinii*.

Specifically, the prevalence of *B. afzelii* among *B. burgdorferi* s.l.-positive *I. ricinus* ticks was highest in the Black Sea region, with statistically significant differences compared to Stara Zagora, where it was lowest (54.17% vs. 30.0%, *p* = 0.041; χ^2^ = 4.027; OR = 0.363; 95% CI: 13.3–91.9%). The prevalence difference between Stara Zagora and the Pleven region was not statistically significant (30.0% versus 40.18%, *p* = 0.215; χ^2^ = 1.536; OR = 0.636; 95% CI: 31.3–130.2%).

When comparing the prevalence of *B. afzelii* among the total *I. ricinus* ticks, the Pleven region presented the highest rate at 21.03%. This was followed by the Black Sea region at 12.5%, with the Stara Zagora region showing the lowest prevalence at 10.64%. The differences in prevalence between the Pleven and Stara Zagora regions are statistically significant (21.03% versus 10.64%; *p* = 0.013; χ^2^ = 6.533; OR = 0.447; 95% CI: 23.9–83.8%).

Furthermore, the prevalence of *B. garinii* is highest among *Borrelia*-positive ticks in the Black Sea region, with significant differences noted when compared to Stara Zagora (37.5% vs. 14.0%, *p* = 0.022; χ^2^ = 5.284, OR = 3.683; 95% CI: 11.6–16.3%). Differences in the rate of infection between Stara Zagora and Pleven regions are also significant (14.0% vs. 30.36%, *p* = 0.027; χ^2^ = 4.893, OR = 2.678; 95% CI: 10.95–65.5%).

The prevalence of *B. garinii* among the total number of *I. ricinus* ticks is highest in Pleven (15.89%), followed by the Black Sea region (8.65%) and lowest in the Stara Zagora region (4.96%). Differences are statistically significant between the Pleven and Stara Zagora regions (*p* = 0.002; χ^2^ = 9.928, OR = 0.277; 95% CI: 11.9–64.3%), and differences between the Stara Zagora and Black Sea regions are statistically insignificant (*p* = 0.248; χ^2^ = 1.335, OR = 0.551 95% CI: 19.8–153.2%).

The prevalence of *I. ricinus* ticks co-infected with both *Borrelia* genospecies followed the same trends, being highest for Pleven (10.28%), followed by the Black Sea region (4.81%), and lowest in the Stara Zagora region (2.13%). Additionally, the rate of coinfection with both *B. afzelii* and *B. garinii* is higher in Kaylaka Park (9.91%) compared to Metodiy Kusev Park (2.47%), but the difference is not statistically significant.

Calculated for Bulgaria as a whole, the overall prevalence of *B. afzelii* (among *B. burgdorferi* s.l.-positive ticks: 39.25%; among total *I. ricinus*: 15.9%) remains higher than that of *B. garinii* (among *B. burgdorferi* s.l.-positive ticks: 26.88%; among total *I. ricinus*: 10.89%).

## 4. Discussion

This study aims to survey the prevalence of *B. afzelii* and *B. garinii* among *Borrelia burgdorferi* s.l.-positive *I. ricinus* ticks from three geographical regions in Bulgaria. This is the first study in Bulgaria to directly compare the prevalence across different geographical regions, including popular recreational and urban green spaces. To date, no research has been conducted on the distribution of *Borrelia* genospecies in infected hard ticks *Ixodes ricinus* in Bulgaria.

We found the significantly highest prevalence of *Borrelia burgdorferi* s.l. in *I. ricinus* ticks collected in the Pleven region compared to those in the Stara Zagora and Black Sea regions in Bulgaria. In comparison to the results from our previous study, we observed a slight increase in the infection rate of *Borrelia burgdorferi* s.l. in adult *Ixodes ricinus* ticks collected from the same region in Pleven [19]. The infection rate rose from 48.53% in 2019 to 53.65% in 2021. Considering these results, we can conclude that the rate of tick infection with *Borrelia burgdorferi* s.l. in this area remains consistently high. Additionally, we observed a significantly higher prevalence of infected ticks with another tick-borne pathogen, *Anaplasma phagocytophilum*, in the Pleven region compared to in the Black Sea Coast region [20]. This steadily higher infection rate with tick-borne pathogens in Kaylaka Park may be attributed to the stable populations of large and small mammals, birds, and reptiles, which serve as reservoir hosts, maintaining the spread of pathogens throughout the life cycle of *I. ricinus* in this region. Although the prevalence of infected ticks is numerically higher in Kaylaka Park (52.97%) than in Metodiy Kusev Park (41.98%), the lack of a statistically significant difference between the two sites indicates a uniformly high epidemiological risk across these major urban green spaces. Epidemiological studies conducted on the prevalence of *B. burgdorferi* s.l. in ticks from neighboring countries have yielded results that are quite similar to ours. Borşan et al. present a study conducted in urban and peri-urban areas of Cluj-Napoca, one of the largest cities in Romania. In their research, they detected *B. burgdorferi* s.l. DNA in 37.9% of *I. ricinus* ticks collected from all sampling sites in 2018 [25]. Similarly, Richtrova et al. reported the highest percentages of infected *I. ricinus* ticks, ranging from 24.9% to 38.5%, in various central urban areas in Prague, Czech Republic, despite the lower diversity of reservoir hosts in these locations [26]. Additionally, an international team compiled a comprehensive review article that summarizes and analyzes species diversity and infection rates of *I. ricinus* ticks in six countries in the Western Balkans, including Serbia and North Macedonia [27]. Authors reported that infection rates of *Borrelia* in *I. ricinus* ticks vary from 8.1% to 54.2% depending on different habitats.

The *B. burgdorferi* s.l. complex includes various genospecies that are responsible for causing LB. Although the known number of genospecies in this complex currently exceeds 20 genospecies of Lyme spirochetes, not all of them are causative agents of LB in humans [28,29]. Of these genospecies, *B. afzelii, B. garinii,* and *B. burgdorferi* s.s. are the confirmed etiological agents of various clinical manifestations of LB in humans, including chronic appearances of the disease. Other genospecies have been found in tissue samples from single cases of LB patients, such as *B. valaisiana*, and the clinical significance of others, like *B. lusitaniae*, was elucidated much later. In Europe, the main pathogenic genospecies for humans are *B. afzelii*, which is primarily maintained by small rodents, and *B. garinii*, which is primarily maintained by birds [5,30,31].

In this study, we assessed the prevalence of two genospecies, *B. afzelii* and *B. garinii*, through PCR amplification of genospecies-specific nucleotide sequences from the *16S rRNA* gene. The results showed significant differences between the regions; however, in each region, the highest prevalence was recorded for *B. afzelii*, followed by *B. garinii,* with the lowest prevalence being that co-infected with both *Borrelia* genospecies. The lowest frequency of *B. afzelii* was recorded in Stara Zagora at 10.64%, followed by the Black Sea region at 12.5%, and the statistically highest frequency was found in Pleven at 21.03%. The significantly highest frequency of *B. garinii* was calculated again for the Pleven region (15.89%), compared to the two other regions. This high co-circulation of both genospecies in Pleven is further supported by the highest frequency of co-infection (double-positive ticks) in this region, recorded at 10.28% of all *I. ricinus* collected. *Borrelia afzelii* was the predominant species in the adult ticks, with a prevalence of 19% among 246 *I. ricinus* ticks, collected in the wooded area of the Sofia region in May 2005, as reported by Gladnishka et al. [19]. Our findings align with the results reported in a study of Borsan et al., conducted in Romania, where seven genospecies of *Borrelia* were identified. The most prevalent genotype was *B. afzelii*, found in 12.6% of the infected ticks, followed by *B. lusitaniae* at 9.5% and *B. garinii* at 7.4%, as determined by qPCR [25].

The ecology of *B. burgdorferi* s.l. genospecies is geographically restricted, and the prevalence in ticks varies widely [31,32], likely depending on the density of competent reservoir hosts, which also varies. Tick larvae are primarily infected by small rodents, birds, and some other vertebrates. Small rodents, as the main inhabitants of wooded areas, serve as the primary reservoir for *B. afzelii*, while birds are the main reservoirs for *B. garinii* [5,33,34]. Each genotype of this pathogen not only prefers specific competent hosts but also exhibits different clinical manifestations in humans, indicating that the eco-epidemiology of LB is extremely complex. For example, *B. afzelii* infection is associated with skin manifestations, while infection with *B. garinii* is mainly manifested with neuroborreliosis, although each genospecies has the potential to affect multiple body systems [35]. In addition, similar distribution patterns for *B. afzelii* and *B. garinii* genospecies for LB patients have been reported [30].

Our results suggest that there may be additional genospecies present in the infected ticks, as the combined frequencies of the two identified genospecies do not account for the total percentage of ticks infected with *B. burgdorferi* s.l. To identify other genotypes, including *B. burgdorferi* sensu stricto (s.s.), *B. valaisiana, B. lusitaniae,* and others, further analyses using quantitative real-time PCR or sequencing methods are necessary.

A limitation of our study is the different methods of tick collection. Ticks were collected from the vegetation by flagging from two city parks (Kaylaka and Metodi Kusev); however, ticks from the Bleak Sea region were removed from stray (Byala town) and domestic (Sveti Vlas town) dogs. The variation in collection methods may affect the PCR identification of tick-borne pathogens, particularly for fully engorged ticks.

The incidence of LB in Europe has significantly increased, as reported by Burn et al., indicating that there are now up to 128,888 cases annually [36]. The authors analyzed LB incidence in 25 European countries from 2005 to 2020 using publicly available surveillance data. Moreover, another surveillance-based study examining incidence trends from 2015 to 2023 reported an average of 132,000 Lyme borreliosis cases annually across 29 European countries [37]. Data from Bulgaria were included in both studies. Although a temporary decrease in LB cases was observed during 2019–2021, this trend reversed from 2021 to 2023. A recent nationwide study on the LB seroprevalence across different regions of Bulgaria found rates ranging from 0.0% to 20.0%, with the highest rates in Northern provinces reaching up to 20% [17].

Collectively, these findings have raised significant public health concerns. Increased incidence of LB depends on the tick rate of infection with *B. burgdorferi* s.l. and their genospecies. Epidemiological surveys give the actual data for the distribution of *Borrelia* pathogens and have potential for improving prevention measures, such as the use of chemical acaricides and others to control tick populations.

Control of *Ixodes ricinus* populations and the prevention of *Borrelia burgdorferi* s.l. transmission cannot be limited solely to chemical treatment with acaricides. Successfully addressing the increasing incidence of LB in Europe requires the implementation of a multifaceted, integrated approach encompassing both primary and secondary prevention. Primary prevention includes ecological control—through green space management (reducing tall grass and shrubs, removing leaf litter), host control (e.g., treating reservoir hosts such as rodents and deer), and personal preventive measures [38,39]. The latter are key and involve the consistent use of repellents, wearing protective clothing, and immediate body checks for ticks after spending time in endemic areas. Therefore, to be effective, future public health campaigns must be directed toward increasing the individual’s self-efficacy through the development of theory-driven interventions that ensure the necessary skills, resources, and confidence for the sustained application of protective measures. Only such a comprehensive approach, combining ecological control with behavioral change, can lead to a sustainable reduction in the risk of *Borrelia* spp. transmission.

## 5. Conclusions

The *Ixodes ricinus* tick is the most widely distributed tick species in Europe, and it is the primary vector for *Borrelia burgdorferi* s.l., the causative agent of Lyme borreliosis. This study evaluated the prevalence of *B. burgdorferi* s.l. along with its genospecies, *B. afzelii* and *B. garinii*, in *I. ricinus* ticks collected from the Black Sea Coast, the Pleven, and Stara Zagora regions in Bulgaria. The finding revealed statistically significant regional variation in infection prevalence. The highest rate of *B. burgdorferi* s.l. infection was found in the Pleven region, followed by Stara Zagora, while the Black Sea Coast exhibited the lowest level. A comparable pattern was noted for both *B. afzelii* and *B. garinii* genospecies, demonstrating the highest occurrence in Pleven, following the Black Sea region, while Stara Zagora showed the lowest prevalence. Overall, in Bulgaria, the frequency of *B. afzelii* is higher than that of *B. garinii* among the infected ticks. These results highlight significant public health concerns regarding Lyme borreliosis in the studied regions of Bulgaria and underscore the potential for enhancing preventive strategies.

## Figures and Tables

**Table 1:** Collection data of ticks by species, developmental stage, and gender.

Region of Bulgaria	Sex and Stage of *I. ricinus* Ticks				*Rhipicephalus* sp.	*Haemaphysalis* sp.
	Female	Male	Nymph	Total		
**Metodiy Kusev Park**	31	24	26	81	18	3
**“Zheleznik”**	25	25	10	60	13	9
**Stara Zagora**	**56**	**49**	**36**	**141**	31	12
**Byala**	46	30	16	92	133	0
**St. Vlas**	8	3	1	12	18	0
**Black Sea Coast**	**54**	**33**	**17**	**104**	151	0
**KaylakaPark**	101	79	22	202	0	0
**Kartozhabene**	5	7	0	12	0	0
**Pleven**	**106**	**86**	**22**	**214**	0	0

**Table 2:** Primers used for molecular identification of *Borrelia burgdorferi* genospecies.

Primers	Target Gene	Target Sequence (5′-3′)	Amplicon Size	Annealing Temperature
Amplification of genospecies-specific sequences of *B. afzelii*				
BAF	*16S rRNA*	GCA TGC AAG TCA AAC GGA	591 bp	46 °C
BAR		ATA TAG TTT CCA ACA TAG C		
Amplification of genospecies-specific sequences of *B. garinii*				
BGF	*16S rRNA*	GGG ATG TAG CAA TAC ATCT	574 bp	44 °C
BGR		ATA TAG TTT CCA ACA TAG T		

**Table 3:** The prevalence of *Ixodes ricinus* ticks with *Borrelia burgdorferi* s.l., according to their sex and stage in studied regions.

Region of Bulgaria	PCR-Positive *Ixodes ricinus* Ticks for *B. burgdorferi* s.l. Identified by nPCR				
	Female	Male	Adults	Nymph	Total
**Metodiy Kusev Park**	16 (51.61%)	8 (33.33%)	24 (43.64%)	10 (38.46%)	34 (41.98%)
**“Zheleznik”**	7 (28.00%)	5 (20.00%)	12 (24.00%)	4 (40.00%)	16 (26.67%)
**Stara Zagora**	**23 (41.07%)**	**13 (26.53%)**	**36 (34.29%)**	**14 (38.86%)**	**50 (35.46%)**
**Byala**	11 (23.91%)	7 (23.33%)	18 (23.68%)	4 (25.00%)	22 (23.91%)
**St. Vlas**	2 (25.00%)	0 (0.00%)	2 (18.18%)	0 (0.00%)	2 (16.67%)
**Black Sea Coast**	**13 (24.07%)**	**7 (21.21%)**	**20 (22.99%)**	**4 (23.53%)**	**24 (23.08%)**
**KaylakaPark**	57 (56.44%)	41 (51.90%)	98 (54.44%)	9 (40.91%)	107 (52.97%)
**Kartozhabene**	2 (66.67%)	3 (42.86%)	5 (41.67%)	0 (0.00%)	5 (41.67%)
**Pleven**	**59 (55.66%)**	**44 (51.16%)**	**103 (53.65%)**	**9 (40.91%)**	**112 (52.34%)**

**Table 4:** The prevalence of *Ixodes ricinus* ticks with *B. afzelii* and *B. garinii* in studied regions.

Region of Bulgaria	PCR-Positive Ticks for *B. afzelii*		PCR-Positive Ticks for *B. garinii*		PCR Double-Positive Ticks	
	*n*/% of Positive	% of All Ticks	*n*/% of Positive	% of All Ticks	*n*/% of Positive	% of All Ticks
**Stara Zagora region**	15/30.0%	10.64%	7/14.00%	4.96%	3/6.00%	2.13%
**Metodiy Kusev Park**	11/32.35%	13.58%	6/17.65%	7.41%	2/5.88%	2.47%
**Black Sea Coast**	13/54.17%	12.50%	9/37.50%	8.65%	5/20.83%	4.81%
**Pleven region**	45/40.18%	21.03%	34/30.36%	15.89%	22/19.64%	10.28%
**Kaylaka Park**	42/39.25%	20.79%	32/29.91%	15.84%	20/18.69%	9.91%
**Total**	**73/39.25%**	**15.90%**	**50/26.88%**	**10.89%**	**30/16.13%**	**6.54%**

## Data Availability

The data presented in this study are available upon request from the corresponding author.

## References

[1] Strnad M., Honig V., Ruzek D., Grubhoffer L., Rego R.O.M. (2017). Europe-wide meta-analysis of *Borrelia burgdorferi* sensu lato prevalence in questing *Ixodes ricinus* ticks. Appl. Environ. Microbiol..

[2] Estrada-Pena A., Cutler S., Potkonjak A., Vassier-Tussaut M., Van Bortel W., Zeller H., Fernández-Ruiz N., Mihalca A.D. (2018). An updated meta-analysis of the distribution and prevalence of *Borrelia burgdorferi* s.l. in ticks in Europe. Int. J. Health Geogr..

[3] Mannelli A., Bertolotti L., Gern L., Gray J. (2012). Ecology of *Borrelia burgdorferi* sensu lato in Europe: Transmission dynamics in multi-host systems, influence of molecular processes and effects of climate change. FEMS Microbiol. Rev..

[4] Kilpatrick A.M., Dobson A.D.M., Levi T., Salkeld D.J., Swei A., Ginsberg H.S., Kjemtrup A., Padgett K.A., Jensen P.M., Fish D. (2017). Lyme disease ecology in a changing world: Consensus, uncertainty and critical gaps for improving control. Philos. Trans. R. Soc. Lond. B Biol. Sci..

[5] Mysterud A., Stigum V.M., Jaarsma R.I., Sprong H. (2019). Genospecies of Borrelia burgdorferi sensu lato detected in 16 mammal species and questing ticks from northern Europe. Sci. Rep..

[6] Gilbert L., Aungier J., Tomkins J.L. (2014). Climate of origin affects tick (*Ixodes ricinus*) host-seeking behavior in response to temperature: Implications for resilience to climate change?. Ecol. Evol..

[7] Estrada-Pena A., Fernández-Ruiz N. (2022). Is composition of vertebrates an indicator of the prevalence of tick-borne pathogens?. Infect. Ecol. Epidemiol..

[8] Hansford K.M., Wheeler B.W., Tschirren B., Medlock J.M. (2022). Questing *Ixodes ricinus* ticks and *Borrelia* spp. in urban green space across Europe: A review. Zoonoses Public. Health.

[9] Voyiatzaki C., Papailia S.I., Venetikou M.S., Pouris J., Tsoumani M.E., Papageorgiou E.G. (2022). Climate Changes Exacerbate the Spread of *Ixodes ricinus* and the Occurrence of Lyme Borreliosis and Tick-Borne Encephalitis in Europe-How Climate Models Are Used as a Risk Assessment Approach for Tick-Borne Diseases. Int. J. Environ. Res. Public Health.

[10] Rizzoli A., Silaghi C., Obiegala A., Rudolf I., Hubálek Z., Földvári G., Plantard O., Vayssier-Taussat M., Bonnet S., Špitalská E. (2014). Ixodes ricinus and its transmitted pathogens in urban and peri-urban areas in Europe: New hazards and relevance for public health. Front. Public Health.

[11] Alkishe A.A., Peterson A.T., Samy A.M. (2017). Climate change influences on the potential geographic distribution of the disease vector tick *Ixodes ricinus*. PLoS ONE.

[12] Le Dortz L.L., Rouxel C., Polack B., Boulouis H., Lagrée A., Deshuillers P.L., Haddad N. (2024). Tick-borne diseases in Europe: Current prevention, control tools and the promise of aptamers. Vet. Parasitol..

[13] Bellato A., Pintore M.D., Catelan D., Pautasso A., Torina A., Rizzo F., Mandola M.L., Mannelli A., Casalone C., Tomassone L. (2021). Risk of tick-borne zoonoses in urban green areas: A case study from Turin, northwestern Italy. Urban For. Urban Green..

[14] Mathews-Martin L., Namèche M., Vourc’h G., Gasser S., Lebert I., Poux V., Barry S., Bord S., Jachacz J., Chalvet-Monfray K. (2020). Questing tick abundance in urban and peri-urban parks in the French city of Lyon. Parasit. Vectors.

[15] Janzén T., Choudhury F., Hammer M., Petersson M., Dinnétz P. (2024). Ticks-public health risks in urban green spaces. BMC Public Health.

[16] Ermenlieva N., Tsankova G., Todorova T.T. (2019). Epidemiological study of Lyme disease in Bulgaria. Cent. Eur. J. Public Health.

[17] Ngoc K., Trifonova I., Gladnishka T., Taseva E., Panayotova E., Vladimirova I., Ivanova V., Kuteva E., Christova I. (2024). Serological Assessment of *Lyme borreliosis* in Bulgaria: A Nationwide Study. Pathogens.

[18] Gladnishka T., Christova I., Trifonova I., Ivanova V., Panayotova E., Taseva E. (2022). Study on ticks removed from patients for infection with Borrelia burgdorferi and their number depending on temperature and precipitation in 2016–2021. Probl. Infect. Parasit. Dis..

[19] Gladnishka T.K., Tasseva E.I., Christova I.S., Nikolov M.A., Lazarov S.P. (2005). Detection of Borrelia burgdorferi sensu lato, Anaplasma phagocytophilum and spotted fever group rickettsiae in ticks from the region of Sofia, Bulgaria (Acari: Parasitiformes: Ixodidae). Eur. Arachnol..

[20] Stanilov I., Blazhev A., Miteva L. (2023). Anaplasma and Ehrlichia Species in Ixodidae Ticks Collected from Two Regions of Bulgaria. Microorganisms.

[21] Blazhev A., Stanilov I., Miteva L.D., Atanasova M., Blazheva S., Stanilova S. (2022). Prevalence of *Borrelia burgdorferi* Sensu Lato in *Ixodes ricinus* Ticks Collected from Kaylaka Park in Pleven, Bulgaria. Microorganisms.

[22] Georgieva G., Gecheva G., Beron P. (2013). Fauna Bulgaria.

[23] Estrada-Peña A., Mihalca A.D., Petney T.N. (2018). Ticks of Europe and North Africa: A Guide to Species Identification.

[24] Santino I., Berlutti F., Pantanella F., Sessa R., del Piano M. (2008). Detection of Borrelia burgdorferi sensu lato DNA by PCR in serum of patients with clinical symptoms of Lyme borreliosis. FEMS Microbiol. Lett..

[25] Borşan S.D., Ionică A.M., Galon C., Toma-Naic A., Peştean C., Sándor A.D., Moutailler S., Mihalca A.D. (2021). High Diversity, Prevalence, and Co-infection Rates of Tick-Borne Pathogens in Ticks and Wildlife Hosts in an Urban Area in Romania. Front. Microbiol..

[26] Richtrová E., Míchalová P., Lukavská A., Navrátil J., Kybicová K. (2022). Borrelia burgdorferi sensu lato infection in Ixodes ricinus ticks in urban green areas in Prague. Ticks Tick. Borne Dis..

[27] Kapo N., Zuber Bogdanović I., Gagović E., Žekić M., Veinović G., Sukara R., Mihaljica D., Adžić B., Kadriaj P., Cvetkovikj A. (2024). Ixodid ticks and zoonotic tick-borne pathogens of the Western Balkans. Parasit. Vectors.

[28] Stanek G., Reiter M. (2011). The expanding Lyme Borrelia complex--Clinical significance of genomic species?. Clin. Microbiol. Infect..

[29] Margos G., Henningsson A.J., Hepner S., Markowicz M., Sing A., Fingerle V., Sing A. (2022). Borrelia Ecology, Evolution, and Human Disease: A Mosaic of Life. Zoonoses: Infections Affecting Humans and Animals.

[30] Waindok P., Schicht S., Fingerle V., Strube C. (2017). Lyme borreliae prevalence and genospecies distribution in ticks removed from humans. Ticks Tick-Borne Dis..

[31] Kelly P.H., Davis J., Tan Y., Marick H.M., Davidson A., Estrada-Peña A., Moïsi J.C., Stark J.H. (2025). Occurrence and prevalence of host-seeking Ixodes ricinus nymphs infected with Borrelia burgdorferi (sensu lato) genospecies in Europe (1999–2022). Curr. Res. Parasitol. Vector-Borne Dis..

[32] Gern L. (2008). Borrelia burgdorferi sensu lato, the agent of Lyme borreliosis: Life in the wilds. Parasite.

[33] Skuballa J., Petney T., Pfäffle M., Oehme R., Hartelt K., Fingerle V., Kimmig P., Taraschewski H. (2012). Occurrence of different *Borrelia burgdorferi* sensu lato genospecies including *B. afzelii*, *B. bavariensis*, and *B. spielmanii* in hedgehogs (*Erinaceus* spp.) in Europe. Ticks Tick Borne Dis..

[34] Koutantou M., Drancourt M., Angelakis E. (2024). Prevalence of Lyme Disease and Relapsing Fever *Borrelia* spp. in Vectors, Animals, and Humans within a One Health Approach in Mediterranean Countries. Pathogens.

[35] Radolf J.D., Strle K., Lemieux J.E., Strle F. (2021). Lyme Disease in Humans. Curr. Issues Mol. Biol..

[36] Burn L., Vyse A., Pilz A., Tran T.M.P., Fletcher M.A., Angulo F.J., Gessner B.D., Moisi J.C., Stark J.H. (2023). Incidence of Lyme borreliosis in Europe: A Systematic Review (2005–2020). Vector-Borne Zoonotic Dis..

[37] Davidson A., Davis J., Brestrich G., Moisi J.C., Jodar L., Stark J.H. (2025). Lyme Borreliosis Incidence Across Europe, 2015-2023: A Surveillance-Based Review and Analysis. Vector Borne Zoonotic Dis..

[38] Corapi K.M., White M.I., Phillips C.B., Daltroy L.H., Shadick N.A., Liang M.H. (2007). Strategies for primary and secondary prevention of Lyme disease. Nature clinical practice. Rheumatology.

[39] Connally N.P., Hornbostel V.L., Dyer M.C., Hojgaard A., Osikowicz L.M., Christopher D.A., Mather T.N. (2025). The impact of deer exclusion fencing on host-seeking blacklegged ticks on suburban residential properties in southern. N. Engl. J. Med. Entomol..

